# The relationship between treatment-related changes in total hip BMD measured after 12, 18, and 24 mo and fracture risk reduction in osteoporosis clinical trials: the FNIH-ASBMR-SABRE project

**DOI:** 10.1093/jbmr/zjae126

**Published:** 2024-08-04

**Authors:** Tatiane Vilaca, Marian Schini, Li-Yung Lui, Susan K Ewing, Austin R Thompson, Eric Vittinghoff, Douglas C Bauer, Richard Eastell, Dennis M Black, Mary L Bouxsein

**Affiliations:** Division of Clinical Medicine, University of Sheffield, Sheffield S5 7AU, United Kingdom; Division of Clinical Medicine, University of Sheffield, Sheffield S5 7AU, United Kingdom; Research Institute, California Pacific Medical Center, San Francisco, CA 94158, United States; Department of Epidemiology and Biostatistics, University of California at San Francisco, San Francisco, CA 94158, United States; Department of Epidemiology and Biostatistics, University of California at San Francisco, San Francisco, CA 94158, United States; Department of Epidemiology and Biostatistics, University of California at San Francisco, San Francisco, CA 94158, United States; Department of Epidemiology and Biostatistics, University of California at San Francisco, San Francisco, CA 94158, United States; Department of Medicine, University of California at San Francisco, San Francisco, CA 94158, United States; Division of Clinical Medicine, University of Sheffield, Sheffield S5 7AU, United Kingdom; Department of Epidemiology and Biostatistics, University of California at San Francisco, San Francisco, CA 94158, United States; Center for Advanced Orthopedic Studies, Beth Israel Deaconess Medical Center and Department of Orthopedic Surgery, Harvard Medical School, Boston, MA 02215, United States

**Keywords:** Bone mineral density, osteoporosis medication, fracture risk reduction, surrogate, meta-regression, randomised controlled trial, vertebral fracture, non-vertebral fracture, clinical fracture, hip fracture

## Abstract

There is a strong association between total hip bone mineral density (THBMD) changes after 24 mo of treatment and reduced fracture risk. We examined whether changes in THBMD after 12 and 18 mo of treatment are also associated with fracture risk reduction. We used individual patient data (*n* = 122 235 participants) from 22 randomized, placebo-controlled, double-blind trials of osteoporosis medications. We calculated the difference in mean percent change in THBMD (active-placebo) at 12, 18, and 24 mo using data available for each trial. We determined the treatment-related fracture reductions for the entire follow-up period, using logistic regression for radiologic vertebral fractures and Cox regression for hip, non-vertebral, “all” (combination of non-vertebral, clinical vertebral, and radiologic vertebral) fractures and all clinical fractures (combination of non-vertebral and clinical vertebral). We performed meta-regression to estimate the study-level association (*r*^2^ and 95% confidence interval) between treatment-related differences in THBMD changes for each BMD measurement interval and fracture risk reduction. The meta-regression revealed that for vertebral fractures, the *r*^2^ (95% confidence interval) was 0.59 (0.19, 0.75), 0.69 (0.32, 0.82), and 0.73 (0.33, 0.84) for 12, 18, and 24 mo, respectively. Similar patterns were observed for hip: *r*^2^ = 0.27 (0.00, 0.54), 0.39 (0.02, 0.63), and 0.41 (0.02, 0.65); non-vertebral: *r*^2^ = 0.27 (0.01, 0.52), 0.49 (0.10, 0.69), and 0.53 (0.11, 0.72); all fractures: *r*^2^ = 0.44 (0.10, 0.64), 0.63 (0.24, 0.77), and 0.66 (0.25, 0.80); and all clinical fractures: *r*^2^ = 0.46 (0.11, 0.65), 0.64 (0.26, 0.78), and 0.71 (0.32, 0.83), for 12-, 18-, and 24-mo changes in THBMD, respectively. These findings demonstrate that treatment-related THBMD changes at 12, 18, and 24 mo are associated with fracture risk reductions across trials. We conclude that BMD measurement intervals as short as 12 mo could be used to assess fracture efficacy, but the association is stronger with longer BMD measurement intervals.

## Graphical Abstract

**Graphical Abstract:**
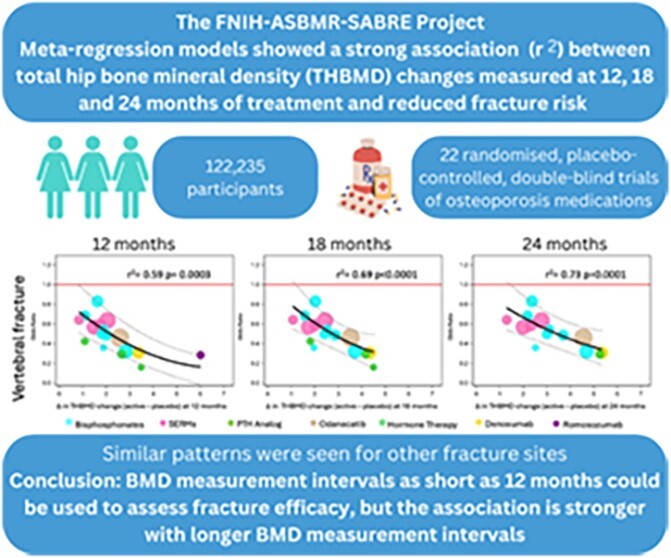

## Introduction

We have shown that changes in total hip bone mineral density (THBMD) after 24 mo of treatment are strongly associated with fracture risk reductions across randomized trials of osteoporosis therapies, regardless of the mechanisms of action. Based on these findings, the treatment-related change in THBMD after 24 mo is an excellent surrogate endpoint for fractures in new clinical trials of osteoporosis therapies.^1^^,^^2^ Several levels of evidence support BMD as a good surrogate marker. First, BMD is strongly associated with whole bone strength in laboratory-based studies of human cadaveric tissue.^3–5^ Second, the treatment-related increase in BMD is plausibly associated with a decrease in the risk of fractures as population-based observational studies reveal a strong and consistent association between higher BMD and lower risk of fractures.^6–9^ Third, meta-regressions using both published^10^ and individual data^1^ from randomized controlled trials of osteoporosis therapies have shown associations between improvements in BMD and reductions in the risk of fractures.

Our previous analyses evaluated the association between the change in THBMD after 24-mo of osteoporosis treatment and fracture risk reductions. The study investigated the efficacy of three different BMD measurement sites (total hip, femoral neck, and lumbar spine) as surrogate outcomes for fracture risk. Meta-regression analyses revealed similar performance among these sites within each fracture type, but changes in total hip and femoral neck BMD were more strongly associated with fracture reduction compared with lumbar spine BMD, which can be confounded by factors such as aortic calcification and degenerative changes, especially in older adults. At the hip, total hip measurements are generally more reproducible than those at the femoral neck, making total hip measurements preferable for future trials focusing on longitudinal changes in BMD.^1^

We also determined the surrogate threshold effect (STE), which corresponds to the magnitude of the treatment effect on the surrogate (ie THBMD change), that would predict a significant treatment effect on the final outcome (ie a reduction in the risk of fractures).^11^^,^^12^ The use of THBMD change as a surrogate endpoint for fracture could reduce the size, duration, and ultimately the cost of trials for new osteoporosis therapies. Despite the clear benefits of reducing osteoporosis drug trials to 24 mo, some drugs might not be used for 24 mo due to mechanistic or safety reasons and shorter trial duration might be desirable. For example, some osteoporosis drugs are used only for 12 mo (eg romozosumab),^13^ whereas other drugs, such as abaloparatide,^14^ were approved based on 18-mo-long trials. However, the relationships between shorter treatment-related changes in BMD and fracture risk reduction are unknown.

Moreover, for the prior analyses of 24-mo changes in BMD, we included data from 81 497 participants enrolled in 16 RCT’s. However, data from 6 trials that did not have 24-mo BMD information were not included.^13–18^ Evaluation of shorter BMD measurement intervals would allow the inclusion of a greater number of trials in the analysis, thereby enhancing the generalizability of the findings.

Thus, the aim of the current analyses was to investigate the relationship between the treatment-related change in THBMD and the risk of fractures over shorter BMD measurement intervals. To do so, we conducted meta-regressions for BMD measurement intervals of 12, 18, and 24 mo. We hypothesized that longer BMD measurement intervals would result in stronger associations with anti-fracture efficacy.

## Materials and methods

### Search strategy and selection criteria

The methodology for the systematic review and meta-regression analysis has been previously described in detail.^1^^,^^2^^,^^10^ Briefly, we aimed to identify and obtain individual patient data (IPD) from all randomized, placebo-controlled trials of osteoporosis medications with fracture outcomes. Using the IPD, we standardized the fracture definitions across all trials. We defined “all fractures” as the combination of non-vertebral, clinical vertebral, and radiographic vertebral fractures; and “all clinical fractures” as the combination of non-vertebral and clinical vertebral fractures. We excluded fractures due to major trauma (ie trauma sufficient to cause a fracture in a young, healthy individual) when reported. In one study, more than half of non-vertebral fractures were originally excluded due to trauma,^19^ whereas we included all non-vertebral fractures from that study. We used the individual study definitions for radiographic vertebral fractures, which were based on comparing the baseline with one or more follow-up lateral spine radiographs. The criteria for identifying radiographic vertebral fractures varied among trials as quantitative morphometry,^20^ semi-quantitative assessment,^21^ or a combination of these criteria were used.

We created standardized BMD (g/cm^2^) values that were comparable across the different dual-energy X-ray absorptiometry devices used in the trials (Hologic, Bedford, MA; GE Lunar, Madison, WI; and Norland Corporation, Fort Atkinson, WI). Specifically, we converted Lunar and Norland BMD values to Hologic BMD values for the total hip, femoral neck, and lumbar spine using previously published methods.^22^^,^^23^ For this analysis, we focused on changes in THBMD.

### Data analysis

We used IPD from placebo-controlled trials for these analyses. When the study reported multiple doses, we combined the active treatment groups, regardless of the dose, except in two trials of risedronate.^24^^,^^25^ In these two trials, patients randomized to 2.5 mg were excluded from follow-up early, and thus, only the 5 mg dose was included in the current analyses. When the study also included an active comparator, the active comparator group was excluded.^14^^,^^26^ Furthermore, trials with 5 or less fractures of a specific type were not included in the meta-regression analysis for that specific fracture type.

We used IPD to estimate the difference (active-placebo) in the mean percent change in THBMD at 12, 18, and 24 mo for each study, when data were available. For 10 studies that did not measure BMD at 18 mo,^19^^,^^27–34^ 18-mo BMD change was estimated as the average of the 12- and 24-mo BMD changes in the IPD dataset. We used percentage change rather than absolute change in total hip BMD because we previously reported that percent BMD changes were as informative as absolute changes.^1^^,^^35^

We also used IPD to estimate the log relative risk (RRs) for incident fractures for active vs. placebo for each study. When time to the fracture was known (hip, non-vertebral, all and all clinical fractures), we used Cox proportional hazard models to estimate the treatment effect on fracture risk reduction within each study, with results outputted as log hazard ratios (log HRs). In contrast, for radiographic vertebral fractures, where time to event was unknown, we used logistic regression to estimate treatment effect on fracture risk reduction, with results outputted as log odds ratios (log ORs). We determined the treatment-related fracture reductions for the entire follow-up period, except for the FRAME study. Is this trial, we only included data from the first 12 mo which is the placebo-controlled period. The association between treatment and fracture risk (eg OR or HR) that we calculated sometimes differed from the original published results due to different fracture definitions, the exclusion of traumatic fractures, or updates to the clinical trial dataset after publication.

For each study, we merged the treatment-related differences in mean percent change in THBMD at 12, 18, and 24 mo with the log RRs for incident fractures for active vs. placebo to create a study-level database. We used this database to perform the meta-regression analyses, using each trial as the unit of analysis. Each study was weighted by the inverse of its standard error of the log RR for the given fracture outcome: study weight = 1/standard error of log RR.

For each BMD measurement interval, we used a weighted linear regression model on the study-level dataset to estimate the association of the active-placebo difference in mean percentage change in THBMD during the given interval with the log HR or log OR for incident fracture for active vs. placebo, weighted by the inverse of its variance. This was implemented in SAS using the generalized linear model (GLM) procedure. The GLM model provided the *r*^2^, with 95% CI, summarizing the variance explained by the association between treatment-related difference in mean percent change in THBMD and fracture risk reduction. To illustrate the results, we plotted the back-transformed ORs or HRs against the active-placebo difference in percentage change in THBMD for each BMD measurement interval. Each trial is represented by a circle of size proportional to the inverse of the variance of the log HR or OR for the given fracture outcome. To a first approximation, the size of each circle is proportional to the number of fractures in that trial. We added the back-transformed fitted regression line with 95% prediction limits to the corresponding plots, plotting the line from the smallest to largest treatment-related THBMD differences observed in these studies. We calculated the STE as previously described,^1^^,^^2^ namely the active-placebo difference in mean THBMD percent change at which the upper 95% prediction limit from the meta-regression intersects an HR or OR = 1.0 (no treatment effect on fracture risk reduction).^11^^,^^12^

All analyses were by intention to treat, without regard to adherence to treatment, and were performed using SAS software (version 9.4, SAS Institute Inc., Cary, NC, USA) and Stata software (version 17, StataCorp. LLC, College Station, TX, USA).

## Results

In the current analyses, we included data from 122 235 participants from 22 randomized, placebo-controlled trials for whom we had information on both THBMD change and incident fractures (Table S1). Data came from the following trials of osteoporosis medications: 10 bisphosphonate,^17^^,^^25^^,^^27–29^^,^^36–40^ 1 odanacatib,^41^ 2 hormone therapy—1 conjugated equine oestrogen^18^ and 1 conjugated equine oestrogen plus medroxyprogesterone acetate,^16^ 3 parathyroid hormone -PTH receptor agonists^14^^,^^15^^,^^19^, 1 denosumab,^31^ 4 selective estrogen receptor modulator,^26^^,^^32–34^ and 1 romosozumab.^13^ The median study follow-up ranged from 12 to 100 mo. The number of trials used in each analysis ranged from 14 to 21, depending on the specific fracture type and BMD measurement interval combination (Table 1).

**Table 1:** Number of studies, participants, and fractures included in the meta-regression analysis comparing BMD measurement intervals of 12, 18, and 24 mo.

		BMD measurement interval		
Fracture outcome		**12 mo**	**18 mo**	**24 mo**
**Vertebral**	# of studies # of participants^a^ # of fractures	17 80 235 4573	16 73 592 4497	14 70 447 4402
**Hip**	# of studies # of participants^a^ # of fractures	18 117 469 1243	15 83 034 843	14 80 502 837
**Non-vertebral**	# of studies # of participants^a^ # of fractures	21 122 016 10 210	17 85 673 6574	15 81 496 6440
**All**	# of studies # of participants^a^ # of fractures	21 122 017 14 739	17 85 674 10 685	15 81 497 10 459
				
**All clinical**	# of studies # of participants^a^ # of fractures	21 122 016 11 496	17 85 673 7495	15 81 496 7353

a Total number of participants used in calculating the OR or HR for each fracture type.

Depending on the fracture type, the 12-mo analyses included 80 235–122 017 participants enrolled in 17–21 trials, adding ~10 000 more participants to the vertebral fracture analysis and 40 000 more participants to the hip, non-vertebral, all, and all clinical analyses than the respective 24-mo analyses. The mean net differences in THBMD changes at 12 mo in the active vs. placebo treatment groups ranged from 0.84% to 6.02% across the studies.

**Figure 1:**
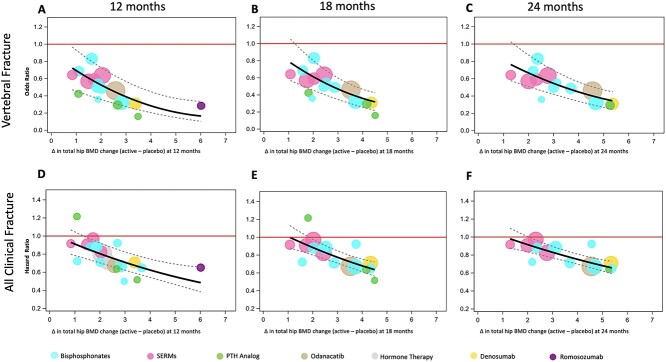
Meta-regression plots displaying the association of active-placebo differences in THBMD percent change and vertebral and all clinical fracture risk reduction for 12- (A, D), 18- (B, E), and 24-mo (C,F) BMD measurement intervals, respectively. Dashed lines represent 95% prediction intervals. Individual trials are represented by circles with areas that are approximately proportional to the number of fractures in the trial. Drugs of the same class are represented by symbols of the same color. The red horizontal line is the odds/hazard ratio of 1.0 (no treatment effect on fracture risk), and the STE is the point where the upper 95% prediction interval intersects this line.

The meta-regression revealed significant associations between the treatment-related difference (active—placebo) in THBMD change at 12 mo and reductions in risk for all fracture types, such that greater gains in THBMD were associated with larger fracture risk reductions (Table 2, Figure 1). The association between the treatment-related difference in THBMD change and fracture risk reduction tended to be stronger for vertebral fractures (*r*^2^ = 0.59, *p*=.0003), than for hip (*r*^2^ = 0.27, *p*=.03), non-vertebral (*r*^2^ = 0.27, *p*=.02), all (*r*^2^ = 0.44, *p*=.001), and all clinical fractures (*r*^2^ = 0.46, *p*=.0007). Illustration of this association is shown in Figure 1 for vertebral and all clinical fractures, whereas graphs for the other fracture types are shown in Figures S1–S3. The STE, the minimum treatment-related increase in THBMD associated with a significant reduction in the risk of fracture, varied by fracture type (Table 3) and was lowest for vertebral fracture (0.84%) and highest for hip fracture (1.76%).

**Table 2:** *r*  ^2^, 95% confidence intervals and *p*-values for the association between treatment-related differences in THBMD change and fracture risk reduction over 12, 18, and 24 mo.

**Fracture outcome**		BMD measurement interval		
		12 mo	18 mo	24 mo
**Vertebral**	*r* ^2^	0.59	0.69	0.73
	95% CI	(0.19, 0.75)	(0.32, 0.82)	(0.33, 0.84)
	*p*-value	.0003	<.0001	.0001
**Hip**	*r* ^2^	0.27	0.39	0.41
	95% CI	(0.00, 0.54)	(0.02, 0.63)	(0.02, 0.65)
	*p*-value	.03	.01	.01
**Non-vertebral**	*r* ^2^	0.27	0.49	0.53
	95% CI	(0.01, 0.52)	(0.10, 0.69)	(0.11, 0.72)
	*p*-value	.02	.002	.002
**All**	*r* ^2^	0.44	0.63	0.66
	95% CI	(0.10, 0.64)	(0.24, 0.77)	(0.25, 0.80)
	*p*-value	.001	.0002	.0002
**All clinical**	*r* ^2^	0.46	0.64	0.71
	95% CI	(0.11, 0.65)	(0.26, 0.78)	(0.32, 0.83)
	*p*-value	.0007	.0001	<.0001

**Table 3:** STE^a^ for BMD measurement intervals of 12, 18, and 24 mo.

Fracture type	BMD measurement interval		
	12 mo	18 mo	24 mo
**Vertebral (%)**	0.84	1.27	1.43
**Hip (%)**	1.76	2.59	3.07
**Non-vertebral (%)**	1.16	1.91	2.13
**All (%)**	1.30	1.65	1.83
**All clinical (%)**	1.37	1.85	2.04

^a^STE is the minimum treatment-related difference (active-placebo) in total hip BMD change required to predict a significant reduction in fracture risk.

The difference (active—placebo) in THBMD change at 12 mo was less than 3.67% for all trials except for the FRAME trial of romosozumab, where the THBMD difference (active—placebo) was 6.02%. However, similar *r*^2^ values were observed in the meta-regression when the FRAME trial was excluded from the analyses: *r*^2^ = 0.64 (*p* = .0002) for vertebral fractures, *r*^2^ = 0.33 (*p* = .01) for hip fractures, *r*^2^ = 0.36 (*p* = .005) for non-vertebral fractures, *r*^2^ = 0.51 (*p* = .0004) for all fractures, and *r*^2^ = 0.56 (*p* = .0002) for all clinical fractures.

The analyses using 18-mo THBMD changes included 73 592−85 892 participants enrolled in 15−17 trials, depending on the fracture type (Table 1). Like the 12-mo analysis, the meta-regression also revealed significant associations between the treatment-related difference (active—placebo) in THBMD change at 18 mo and reductions in risk for all fracture types (Table 2, Figure 1). The *r*^2^ values for the 18-mo analyses were highest for vertebral, all, and all clinical fractures (*r*^2^ = 0.63–0.69). As expected, the STE values at 18 mo were higher than for the 12-mo analysis (Table 3).

The meta-regression results using 24-mo treatment-related differences in THBMD change, several of which were previously reported,^1^ reveal moderate to strong associations with fracture risk reductions (Table 2, Figure 1). Specifically, the treatment-related difference in THBMD changes were associated with reductions in risk for vertebral (*r*^2^ = 0.73, *p* = .0001), hip (*r*^2^ = 0.41, *p* = .01), and non-vertebral fractures (*r*^2^ = 0.53, *p* = .002). In the current analysis, we have also included all fractures (*r*^2^ = 0.66, *p* = .0002) and all clinical fractures (*r*^2^ = 0.71, *p* < .0001). The STE values at 24 mo were higher than values for 12 and 18 mo: 1.43% for vertebral, 3.07% for hip, 2.13% for non-vertebral, 1.83% for all fractures, and 2.04% for all clinical fractures (Table 3).

## Discussion

Previously, we showed that treatment-related differences in in total hip BMD change at 24 mo were associated with reductions in fracture risk. In the current analyses, we expanded this work, aiming to determine whether treatment-related differences in total hip BMD change over shorter intervals would also be associated with fracture risk reductions. Using meta-regressions based on IPD, we found significant associations between treatment-related differences in total hip BMD change at 12, 18, and 24 mo and reductions in vertebral, hip, non-vertebral, all, and all clinical fractures. We observed stronger associations, as measured by greater *r*^2^ values, with longer BMD measurement intervals, though *r*^2^ values were largely similar at 18 and 24 mo, and the 95% confidence interval for *r*^2^ for the three timepoints overlapped.

Not surprisingly, the STEs were also smaller with shorter BMD intervals since the magnitude of treatment-related differences in BMD changes is lower with shorter measurement intervals. Proportional hazards in Cox regression entail a constant relative risk of an event over time. This implies that the hazard function for any two groups (placebo and active drug) remains proportional throughout the observation period. Conversely, treatment-related differences in BMD changes are not linear over time. For this reason, at the 12-mo time point, the STE was more than half that at 24 mo, as the increase in BMD in the second year is often less than in the first year.^27^^,^^36^^,^^40^

Whereas there is no established threshold to define the *r*^2^ value from the meta-regression required to establish that a surrogate endpoint is valid, the literature suggests that an *r*^2^ value *>*0.65 is an appropriate target for the acceptability of a surrogate endpoint.^11^ Our results indicate that longer BMD measurement intervals increase the likelihood of the *r*^2^ value reaching this threshold. For example, the *r*^2^ value from the meta-regression was close to or exceeded this threshold at the 18- and 24-mo BMD intervals for vertebral, all, and all clinical fractures (*r*^2^ = 0.63–0.71).

However, longer studies might not be feasible or desirable for drugs whose effects are optimized by or restricted to shorter periods. For example, romosozumab is licensed to be used only for 12 mo as that is when it showed anti-fracture efficacy. In addition, trials of PTH analogs were shorter than 24 mo due to safety concerns regarding osteosarcoma, based on animal studies. Furthermore, there are ethical concerns about longer duration placebo-controlled trials in individuals at moderate or high risk for fracture. The current analysis provides evidence that the association between THBMD change and fracture risk reduction due to treatment is present as early as 12 mo, although the *r*^2^ values were close to the desirable threshold only for vertebral fracture (*r*^2^ = 0.59).

Alternative approaches for trial design using BMD as a surrogate endpoint when it is desirable to only use the “test” drug for 18 mo or less could include sequential treatment. This approach has been used in several recent phase III trials.^13^^,^^42^^,^^43^ For example, in the FRAME trial, romosozumab was compared with placebo for the first 12 mo, whereafter both groups received denosumab for an additional 12 mo. A longer BMD interval could then be used to estimate the comparative efficacy of the sequential treatment.

The current analyses included osteoporosis drugs with several mechanisms of action, showing that the use of THBMD change as a surrogate endpoint for fracture risk reduction applies across all categories of osteoporosis drugs. However, the optimum observation time might need to be adjusted for the particularities of each drug since the mechanisms of action are different. Some drugs might need to be used longer to positively affect BMD, and therefore, very short observation periods might not be adequate. As mentioned above, other drugs might have their use limited by safety concerns, making longer observation periods not desirable or possible. Finally, some drugs might have limited periods of action and require specific follow-up periods. Therefore, different trial durations and/or designs might be required in selected cases. Having flexibility to choose a BMD measurement interval that is most appropriate given the specific characteristics of the new drug treatment is a positive aspect of using THBMD change as a surrogate endpoint. We included the data from the initial 12 mo of the FRAME trial, when romosozumab was compared with placebo. Romosozumab showed an increase in THBMD 64% higher than the second-highest increase observed. Despite this greater increase, the romosozumab trial still fell within the 95% prediction limits of the meta-regression curve. Because extreme values can have an impact on regression analyses, we conducted a sensitivity analysis excluding the FRAME trial. The *r*^2^ for vertebral, hip, and non-vertebral fractures was numerically greater with the inclusion of romosozumab, while for all, and all clinical fractures, the *r*^2^ was numerically smaller, but overall, the results observed were similar with and without data from the FRAME trial providing evidence that the observed association was not unduly influenced by this study. This also illustrates how the inclusion of drugs with varying mechanisms of action might increase the variability of the results since the data are less homogeneous, but it also enhances the results’ generalizability.

For each BMD measurement interval, there were different sets of studies due to the variable timing of BMD measurements and availability of fracture assessments in each of the studies included. It would be desirable to compare the *r*^2^ for 12, 18, and 24 mo in the same set of studies, to establish the most appropriate BMD measurement interval. However, this approach would limit the comparison to the set of studies from which we have data on 24-mo BMD and thereby exclude several trials of osteoanabolic therapies. The analyses at 12 and 18 mo include more studies, but the set of studies is different, and thus, direct comparisons of the *r*^2^ values between the three BMD measurement intervals must be interpreted carefully.

This study has several strengths. We used a comprehensive database of IPD from RCTs following standardized criteria for the analysis of total hip BMD and fracture definition. We analysed the association between BMD change and fracture reduction for three different BMD measurement intervals, allowing for the particularities of the study design and drugs’ mechanisms of action. The analysis of shorter BMD measurement intervals allowed the inclusion of additional studies that did not have BMD measurements at 24 mo. Because the power and precision of these regressions depend on the number studies included, the increase in the number of trials included makes the analyses more robust. Notably, only one osteoanabolic drug (teriparatide) was included in the 24-mo analyses, but there are four included in the 12-mo analysis, three PTH analogs and romosozumab, enhancing the generalizability to the findings. Moreover, the current analyses expanded our prior work by including a new fracture category, namely “all clinical” that may be useful for trial sponsors and regulatory agencies.

This study also has limitations. For several studies THBMD was not measured at 18 mo, and therefore, we estimated 18-mo BMD change based on the 12- and 24-mo measurements. The trials included in these analyses were all placebo-controlled mono-therapy trials, and further analyses are needed to demonstrate the association between BMD changes and fracture reduction in trials of combination/sequential treatment and in trials with an active comparator. Trials of anabolic drugs were smaller, had a relatively shorter follow-up, and were not powered to show an effect on hip fractures. Thus, most of the data on hip fractures come from bigger and longer trials of anti-resorptive drugs. Thus, hip fracture outcomes from longer trials of anabolic drugs would be helpful; however, these data are currently not available, as the trials have not been conducted. Few of the studies included men, and thus, sex-stratified analyses were not possible. However, BMD-bridging studies are routinely used to assess and approve osteoporosis drugs in men, as there is no indication that the association between the treatment-related change in BMD and reduction in fracture differs by sex.

Notably, these results cannot be applicable to individual patients since the analyses aim to assess the ability of changes in BMD to serve as a surrogate endpoint for fractures for future clinical trials, and thereby, they focus on relative risk reductions between the groups. This would not be appropriate to estimate absolute risk reductions in individuals. In addition, we used total hip BMD to assess treatment-related changes in BMD, but we acknowledge that osteoporosis diagnosis by DXA should be based on the lowest BMD site measured, and treatment decisions should be guided by fracture risk estimates.

It is important to note that additional guidance may be implemented by regulatory agencies to allow a new drug to advance to a registration trial using BMD as a surrogate endpoint for fracture. For example, the new drug will likely need to be tested in preclinical studies, including demonstrating that drug treatment maintains the association between bone mass and bone strength. Clinical studies incorporating iliac crest biopsies may also be required to ensure normal bone histopathology following treatment. In addition, the STE defines the change in BMD that would predict any decrease in the risk of fractures with 95% certainty, but it is possible that regulatory agencies would require a given decrease in the risk of fracture and, consequently, greater increases in BMD. For example, for spine fractures, the STE for 24 mo was 1.4%, but a 30% decrease in the risk of spine fracture would demand a 3.0% increase in THBMD.^2^

In conclusion, we found significant associations between treatment-related differences in total hip BMD change and fracture risk reductions for BMD measurement intervals shorter than 24 mo. These observations, based on robust IPD from randomized placebo-controlled trials, suggest that, when needed, BMD measurement intervals shorter than 24-mo could be used as a surrogate endpoint for fracture outcomes in future clinical trials. This flexibility could be particularly helpful for new treatments that may be administered for a limited period due to efficacy and/or safety considerations. Furthermore, shorter trial durations could lessen the time it takes for new therapies to be available to patients. Ultimately, regulatory agencies will decide under what circumstances a shorter BMD measurement interval would be allowed.

## Supplementary Material

Vilaca_BMD_interval_Supplementary_materials_May24_zjae126

## Data Availability

All study data were acquired by requesting IPD from study sponsors. An overarching data use agreement was created between all parties and individual data use agreements were created between individual study sponsors, FNIH, and University of California, San Francisco (UCSF). Per the data sharing agreements that we have with each sponsor, the data can be used for surrogate marker analyses, including any surrogate qualification processes with regulatory authorities. However, other uses of the data are restricted by this agreement, and UCSF is not allowed to share the data.
