# Supplementary material for: The relationship between treatment-related changes in total hip BMD measured after 12, 18, and 24 mo and fracture risk reduction in osteoporosis clinical trials: the FNIH-ASBMR-SABRE project
Source: J Bone Miner Res. 2024 Aug 4;39(10):1434–42. doi: 10.1093/jbmr/zjae126 (PMC11425700; doi:10.1093/jbmr/zjae126)
Supplement: Vilaca_BMD_interval_Supplementary_materials_May24_zjae126 [file vilaca_bmd_interval_supplementary_materials_may24_zjae126.docx]

Supplementary materials

| **Supplementary Table 1**. Studies included in the meta-regression analyses using BMD measurement intervals of 12, 18, and 24 months | | | | | | | | | | | | | | | |
| --- | --- | --- | --- | --- | --- | --- | --- | --- | --- | --- | --- | --- | --- | --- | --- |
|  | 12 Month | | | | | 18 Month | | | | | 24 Month | | | | |
| Study | V | H | NV | A | AC | V | H | NV | A | AC | V | H | NV | A | AC |
| **Bisphosphonates** | | | | | | | | | | | | | | | |
| ALN Phase 3 (1) |  |  | X | X | X |  |  | X | X | X |  |  | X | X | X |
| FIT VF (2) | X | X | X | X | X | X* | X* | X* | X* | X* | X | X | X | X | X |
| FIT CF (3) | X | X | X | X | X | X* | X* | X* | X* | X* | X | X | X | X | X |
| FOSIT (4) |  |  | X | X | X |  |  |  |  |  |  |  |  |  |  |
| MENS (5) | X |  |  |  |  | X |  |  |  |  | X |  |  |  |  |
| BONE (6) | X | X | X | X | X | X | X | X | X | X | X | X | X | X | X |
| IBAN IV (7) | X | X | X | X | X | X | X | X | X | X | X | X | X | X | X |
| VERT-NA (8) | X | X | X | X | X | X | X | X | X | X | X | X | X | X | X |
| HORIZON PFT (9) | X | X | X | X | X | X* | X* | X* | X* | X* | X | X | X | X | X |
| HORIZON RFT (10) |  | X | X | X | X |  | X* | X* | X* | X* |  | X | X | X | X |
| **Odanacatib** | | | | | | | | | | | | | | | |
| LOFT (11) | X | X | X | X | X | X* | X* | X* | X* | X* | X | X | X | X | X |
| **PTH analog** | | | | | | | | | | | | | | | |
| ACTIVE (12) | X |  | X | X | X | X |  | X | X | X |  |  |  |  |  |
| TOP (13) | X | X | X | X | X | X | X | X | X | X |  |  |  |  |  |
| FPT (14) | X | X | X | X | X | X* | X* | X* | X* | X* | X | X | X | X | X |
| **Estrogen therapy** | | | | | | | | | | | | | | | |
| WHI-E (15) |  | X | X | X | X |  |  |  |  |  |  |  |  |  |  |
| WHI-E+P (16) |  | X | X | X | X |  |  |  |  |  |  |  |  |  |  |
| **Denosumab** | | | | | | | | | | | | | | | |
| FREEDOM (17) | X | X | X | X | X | X* | X* | X* | X* | X* | X | X | X | X | X |
| **Selective Estrogen Receptor Modulator (SERM)** | | | | | | | | | | | | | | | |
| GENERATIONS (18) | X | X | X | X | X | X* | X* | X* | X* | X* | X | X | X | X | X |
| BZA (19) | X | X | X | X | X | X | X | X | X | X | X | X | X | X | X |
| PEARL (20) | X | X | X | X | X | X* | X* | X* | X* | X* | X | X | X | X | X |
| MORE (21) | X | X | X | X | X | X* | X* | X* | X* | X* | X | X | X | X | X |
| **Romosozumab** | | | | | | | | | | | | | | | |
| FRAME (22) | X | X | X | X | X |  |  |  |  |  |  |  |  |  |  |
| **Total number of studies included in each analysis** | **17** | **18** | **21** | **21** | **21** | **16** | **15** | **17** | **17** | **17** | **14** | **14** | **15** | **15** | **15** |
| X, data available and included in analyses; V, vertebral; H, hip; NV, non-vertebral; A, all; AC, all clinical  * For studies which did not measure total hip BMD at 18 months, 18-month BMD change was estimated as the average of the 12-month change and 24-month BMD change in the individual patient dataset; these results were then used to calculate study-level treatment-related difference in mean percent change in total hip BMD at 18 months. | | | | | | | | | | | | | | | |

Supplementary figures

Supplementary figure 1


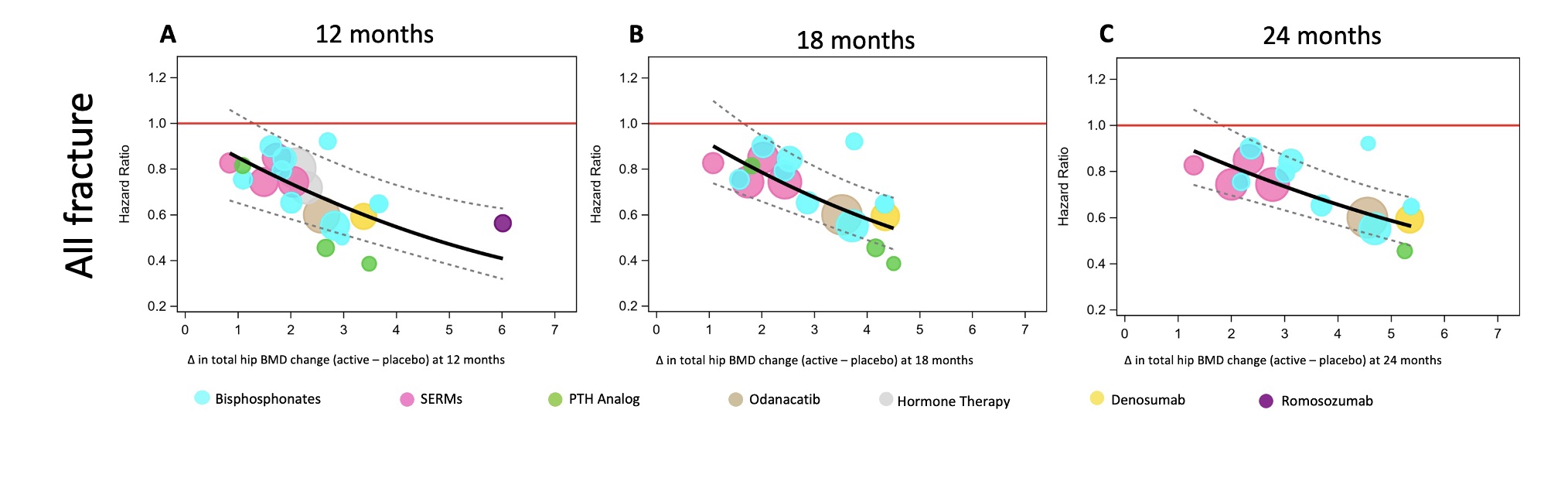


Supplementary Figure 1: Meta-regression and prediction limit plots showing the association of between-treatment differences in THBMD percent change and all fracture risk reduction for 12- (**A**), 18- (**B**), and 24-month (**C**) BMD measurement intervals. Individual trials are represented by circles with areas that are approximately proportional to the number of fractures in the trial. Drugs of the same class are represented by symbols of the same colour. The red horizontal line is the ratio of 1 (no treatment effect), and the STE is the point where the upper 95% prediction limit intersects this line.

Supplementary figure 2


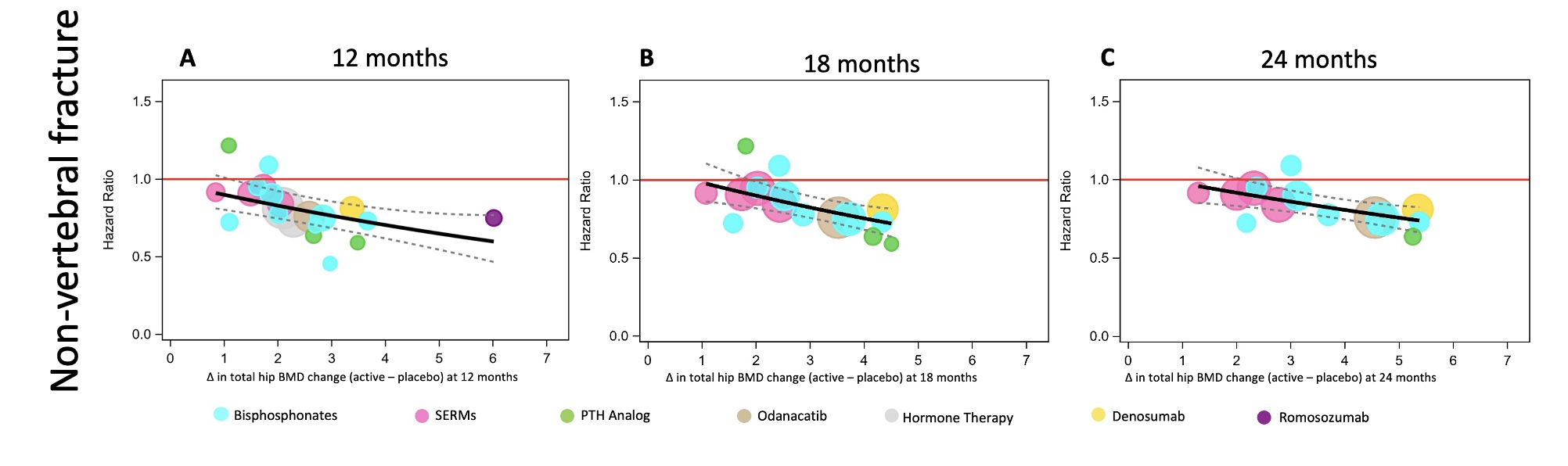


Supplementary Figure 2: Meta-regression and prediction limit plots showing the association of between-treatment differences in THBMD percent change and non-vertebral fracture risk reduction for 12- (**A**), 18- (**B**), and 24-month (**C**) BMD measurement intervals. Individual trials are represented by circles with areas that are approximately proportional to the number of fractures in the trial. Drugs of the same class are represented by symbols of the same colour. The red horizontal line is the ratio of 1 (no treatment effect), and the STE is the point where the upper 95% prediction limit intersects this line.

Supplementary figure 3


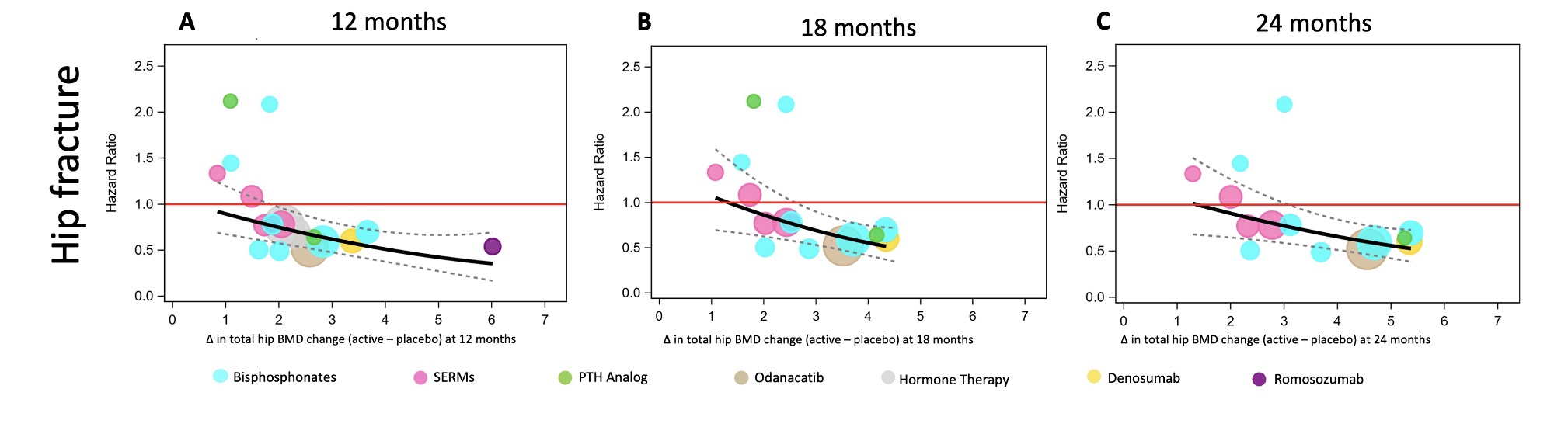


Supplementary Figure 3: Meta-regression and prediction limit plots showing the association of between-treatment differences in THBMD percent change and hip fracture risk reduction for 12- (**A**), 18- (**B**), and 24-month (**C**) BMD measurement intervals. Individual trials are represented by circles with areas that are approximately proportional to the number of fractures in the trial. Drugs of the same class are represented by symbols of the same colour. The red horizontal line is the ratio of 1 (no treatment effect), and the STE is the point where the upper 95% prediction limit intersects this line.

References

1. Liberman UA, Weiss SR, Broll J, Minne HW, Quan H, Bell NH, et al. Effect of oral alendronate on bone mineral density and the incidence of fractures in postmenopausal osteoporosis. The Alendronate Phase III Osteoporosis Treatment Study Group. The New England journal of medicine. 1995;333(22):1437-43.

2. Black DM, Cummings SR, Karpf DB, Cauley JA, Thompson DE, Nevitt MC, et al. Randomised trial of effect of alendronate on risk of fracture in women with existing vertebral fractures. Fracture Intervention Trial Research Group. Lancet. 1996;348(9041):1535-41.

3. Cummings SR, Black DM, Thompson DE, Applegate WB, Barrett-Connor E, Musliner TA, et al. Effect of alendronate on risk of fracture in women with low bone density but without vertebral fractures: results from the Fracture Intervention Trial. Jama. 1998;280(24):2077-82.

4. Pols HA, Felsenberg D, Hanley DA, Stepan J, Munoz-Torres M, Wilkin TJ, et al. Multinational, placebo-controlled, randomized trial of the effects of alendronate on bone density and fracture risk in postmenopausal women with low bone mass: results of the FOSIT study. Fosamax International Trial Study Group. Osteoporosis international : a journal established as result of cooperation between the European Foundation for Osteoporosis and the National Osteoporosis Foundation of the USA. 1999;9(5):461-8.

5. Orwoll E, Ettinger M, Weiss S, Miller P, Kendler D, Graham J, et al. Alendronate for the treatment of osteoporosis in men. NEnglJMed. 2000;343(9):604-10.

6. Chesnut CH, 3rd, Skag A, Christiansen C, Recker R, Stakkestad JA, Hoiseth A, et al. Effects of oral ibandronate administered daily or intermittently on fracture risk in postmenopausal osteoporosis. J Bone Miner Res. 2004;19(8):1241-9.

7. Recker R, Stakkestad JA, Chesnut CH, 3rd, Christiansen C, Skag A, Hoiseth A, et al. Insufficiently dosed intravenous ibandronate injections are associated with suboptimal antifracture efficacy in postmenopausal osteoporosis. Bone. 2004;34(5):890-9.

8. Harris ST, Watts NB, Genant HK, McKeever CD, Hangartner T, Keller M, et al. Effects of risedronate treatment on vertebral and nonvertebral fractures in women with postmenopausal osteoporosis: a randomized controlled trial. Vertebral Efficacy With Risedronate Therapy (VERT) Study Group. Jama. 1999;282(14):1344-52.

9. Black DM, Delmas PD, Eastell R, Reid IR, Boonen S, Cauley JA, et al. Once-yearly zoledronic acid for treatment of postmenopausal osteoporosis. N Engl J Med. 2007;356(18):1809-22.

10. Lyles KW, Colón-Emeric CS, Magaziner JS, Adachi JD, Pieper CF, Mautalen C, et al. Zoledronic Acid and Clinical Fractures and Mortality after Hip Fracture. New England Journal of Medicine. 2007;357(18):1799-809.

11. McClung MR, O'Donoghue ML, Papapoulos SE, Bone H, Langdahl B, Saag KG, et al. Odanacatib for the treatment of postmenopausal osteoporosis: results of the LOFT multicentre, randomised, double-blind, placebo-controlled trial and LOFT Extension study. Lancet Diabetes Endocrinol. 2019;7(12):899-911.

12. Miller PD, Hattersley G, Riis BJ, Williams GC, Lau E, Russo LA, et al. Effect of Abaloparatide vs Placebo on New Vertebral Fractures in Postmenopausal Women With Osteoporosis: A Randomized Clinical Trial. Jama. 2016;316(7):722-33.

13. Greenspan SL, Bone HG, Ettinger MP, Hanley DA, Lindsay R, Zanchetta JR, et al. Effect of recombinant human parathyroid hormone (1-84) on vertebral fracture and bone mineral density in postmenopausal women with osteoporosis: a randomized trial. Ann Intern Med. 2007;146(5):326-39.

14. Neer RM, Arnaud CD, Zanchetta JR, Prince R, Gaich GA, Reginster JY, et al. Effect of parathyroid hormone (1-34) on fractures and bone mineral density in postmenopausal women with osteoporosis. N Engl J Med. 2001;344(19):1434-41.

15. Jackson RD, Wactawski-Wende J Fau - LaCroix AZ, LaCroix Az Fau - Pettinger M, Pettinger M Fau - Yood RA, Yood Ra Fau - Watts NB, Watts Nb Fau - Robbins JA, et al. Effects of conjugated equine estrogen on risk of fractures and BMD in postmenopausal women with hysterectomy: results from the women's health initiative randomized trial. J Bone Miner Res. 2006;21(6):817-28.

16. Cauley JA, Robbins J, Chen Z, Cummings SR, Jackson RD, LaCroix AZ, et al. Effects of estrogen plus progestin on risk of fracture and bone mineral density: the Women's Health Initiative randomized trial. Jama. 2003;290(13):1729-38.

17. Cummings SR, San Martin J, McClung MR, Siris ES, Eastell R, Reid IR, et al. Denosumab for prevention of fractures in postmenopausal women with osteoporosis. N Engl J Med. 2009;361(8):756-65.

18. Cummings SR, McClung M, Reginster JY, Cox D, Mitlak B, Stock J, et al. Arzoxifene for prevention of fractures and invasive breast cancer in postmenopausal women. J Bone Miner Res. 2011;26(2):397-404.

19. Silverman SL, Christiansen C, Genant HK, Vukicevic S, Zanchetta JR, de Villiers TJ, et al. Efficacy of bazedoxifene in reducing new vertebral fracture risk in postmenopausal women with osteoporosis: results from a 3-year, randomized, placebo-, and active-controlled clinical trial. J Bone Miner Res. 2008;23(12):1923-34.

20. Cummings SR, Ensrud K, Delmas PD, LaCroix AZ, Vukicevic S, Reid DM, et al. Lasofoxifene in postmenopausal women with osteoporosis. N Engl J Med. 2010;362(8):686-96.

21. Ettinger B, Black DM, Mitlak BH, Knickerbocker RK, Nickelsen T, Genant HK, et al. Reduction of vertebral fracture risk in postmenopausal women with osteoporosis treated with raloxifene: results from a 3-year randomized clinical trial. Multiple Outcomes of Raloxifene Evaluation (MORE) Investigators. Jama. 1999;282(7):637-45.

22. Cosman F, Crittenden DB, Adachi JD, Binkley N, Czerwinski E, Ferrari S, et al. Romosozumab Treatment in Postmenopausal Women with Osteoporosis. N Engl J Med. 2016;375(16):1532-43.
